# Finding exonic islands in a sea of non-coding sequence: splicing related constraints on protein composition and evolution are common in intron-rich genomes

**DOI:** 10.1186/gb-2008-9-2-r29

**Published:** 2008-02-07

**Authors:** Tobias Warnecke, Joanna L Parmley, Laurence D Hurst

**Affiliations:** 1 Department of Biology and Biochemistry, University of Bath, Claverton Down, Bath, BA2 7AY, UK

## Abstract

**Background:**

In mammals, splice-regulatory domains impose marked trends on the relative abundance of certain amino acids near exon-intron boundaries. Is this a mammalian particularity or symptomatic of exonic splicing regulation across taxa? Are such trends more common in species that *a priori *have a harder time identifying exon ends, that is, those with pre-mRNA rich in intronic sequence? We address these questions surveying exon composition in a sample of phylogenetically diverse genomes.

**Results:**

Biased amino acid usage near exon-intron boundaries is common throughout the metazoa but not restricted to the metazoa. There is extensive cross-species concordance as to which amino acids are affected, and reduced/elevated abundances are well predicted by knowledge of splice enhancers. Species expected to rely on exon definition for splicing, that is, those with a higher ratio of intronic to coding sequence, more introns per gene and longer introns, exhibit more amino acid skews. Notably, this includes the intron-rich basidiomycete *Cryptococcus neoformans*, which, unlike intron-poor ascomycetes (*Schizosaccharomyces pombe*, *Saccharomyces cerevisiae*), exhibits compositional biases reminiscent of the metazoa. Strikingly, 5 prime ends of nematode exons deviate radically from normality: amino acids strongly preferred near boundaries are strongly avoided in other species, and vice versa. This we suggest is a measure to avoid attracting *trans*-splicing machinery.

**Conclusion:**

Constraints on amino acid composition near exon-intron boundaries are phylogenetically widespread and characteristic of species where exon localization should be problematic. That compositional biases accord with sequence preferences of splice-regulatory proteins and are absent in ascomycetes is consistent with selection on exonic splicing regulation.

## Abstract 2

Biased usage of amino acids near exon-intron boundaries is phylogenetically widespread and characteristic of species for which there are expected to be problems defining exons.

## Background

The maxim that 'form follows function', dogmatically adhered to in some early 20th century design and architecture, refers to the idea that the final function of a product should be the only determinant of its design. Phenotypic products of evolutionary processes have also frequently been analyzed in this seductively simple framework.

However, costs of production, the availability of raw materials, and other factors regularly lead to marketable goods being suboptimally designed as far as their immediate function is concerned. Likewise, in, for example, mammals, amino acid content of a protein reflects localized GC content [1].

The need to encode, in exonic sequence, information relevant for correct splicing is another factor with the potential to influence protein composition [2]. Located in the exonic parts of primary mRNA transcripts, exonic splicing enhancers (ESEs) are short (6-8 nucleotides) nucleotide motifs that have been established as a core component of the pre-mRNA splicing mechanism in metazoans [3]. Playing a critical role in constitutive as well as alternative splicing [4], they function at multiple stages of spliceosome assembly by interacting with corresponding RNA recognition motifs in a number of different *trans*-factors [4]. We will primarily focus on SR (serine-arginine) proteins because their binding specificities and functions in splicing regulation have been most extensively characterized. SR proteins appear critical for establishing, in conjunction with other proteins, cross-exon complexes that enable faithful communication between splice sites [3].

Recognition of exonic alongside intronic sequence motifs has been proposed to be pivotal in organisms where a majority of exons are flanked by much larger introns, allowing exons to be efficiently identified and not lost in a sea of intronic sequence [5]. Furthermore, whereas in *Saccharomyces cerevisiae *splice sites and branch point sequences show a high degree of conservation to ensure the intron is correctly targeted by the splicing machinery, these recognition motifs tend to be less well conserved in multicellular organisms [6] and intron-rich fungal genomes [7].

Experimentally raising the number of natural exonic enhancer sites leads to an additive increase in splicing activity [8]. Importantly, ESEs function in a position-dependent manner, their efficiency in catalyzing splicing decreasing with increasing distance from the splice site [9,10]. The significant enrichment near exon-intron boundaries for GAA (a codon known to be overrepresented in ESEs) compared with the synonymous GAG is consistent with this finding [10,11]. More generally, in mammals codons enriched in ESEs are more common near intron-exon boundaries [12].

A recent study by Parmley *et al*. [2] suggests ESEs have also left an imprint on the amino acid composition of proteins. Exploring exonic sequences adjacent to exon-intron boundaries in human and mouse, the authors reported marked trends in the relative abundance of certain amino acids when one moves away from the boundary. Some amino acids, such as lysine (K) and isoleucine (I), are strongly preferred near boundaries whereas others, such as proline (P) and alanine (A), are significantly avoided (for a full list see Tables 1 and 2). This is the case for both 5' and 3' ends of exons. Considering separately the two-fold and four-fold blocks of the six-fold degenerate amino acids, the authors also showed that these trends are owing to avoidances/preferences at the nucleotide level and that there is a high degree of correspondence between the codons preferred and their involvement in computationally predicted and experimentally verified ESEs.

**Table 1:** Amino acids significantly preferred (-) or avoided (+) at 3' ends of exons across species

Amino acids^*†^																							
	
A	C	D	E	F	G	H	I	K	L4	L2	M	N	P	Q	R4	R2	S4	S2	T	V	W	Y	Species (number of exons)^‡^
+_3_		-_7_		-_3_			-_2_	-_1_		-_5_		-_6_	+_2_		+_1_	-_4_		+_4_					Human (178,438)
+_3_		-_6_		-_3_			-_2_	-_1_		-_5_		-_4_	+_1_		+_2_	-_7_	+_5_	+_4_					Mouse (126,268)
		-_4_		-_5_	+_3_		-_1_	-_2_				-_6_	+_2_		+_1_	-_3_				+_4_			*D. rerio* (41,264)
			+_4_	-_1_	+_3_	-_6_	-_2_		+_5_			-_3_			+_1_	-_4_	+_2_	-_5_					*C. elegans *(79,958)
		-_6_	+_3_	-_2_	+_4_	-_8_	-_3_		+_5_	-_5_		-_1_	+_6_		+_2_	-_7_	+_1_	-_4_					*C. briggsae *(74,178)
							-_1_			-_3_		-_2_	+_2_		+_1_	-_4_							*A. gambiae *(7,930)
				-_2_	+_1_		-_1_			-_3_				+_2_									*D. melanogaster *(48,933)
				-_2_	+_1_	-_5_	-_1_		-_4_	+_5_			+_3_		+_2_			+_6_		+_4_		-_3_	*A. mellifera *(45,426)
					+_2_		-_2_		-_1_						-_3_	+_3_				+_1_			*A. thaliana *(109,900)
																							*S. pombe *(2,403)
										-_1_													*S. cerevisiae *(417)

*Indices signify rank order of slope coefficients, separately for negative and positive trends. ^†^L2, R2, S2 and L4, R4, S4 signify the two-fold and four-fold degenerate blocks of leucine, arginine, and serine, respectively. ^‡^*S. cerevisiae *terminal exons were retained given the small number of genes with more than one intron (eight).

**Table 2:** Amino acids significantly preferred (-) or avoided (+) at 5' ends of exons across species

Amino acids^*†^																							
	
A	C	D	E	F	G	H	I	K	L4	L2	M	N	P	Q	R4	R2	S4	S2	T	V	W	Y	Species (number of exons)^‡^
+_2_			-_4_	-_5_		+_7_	-_3_	-_1_		-_2_	-_8_	-_6_	+_1_	+_4_	+_3_	-_7_		+_5_	+_6_				Human (178,438)
+_2_			-_4_	-_5_		+_7_	-_3_	-_1_		-_2_	-_7_	-_6_	+_1_	+_4_	+_3_			+_5_	+_6_				Mouse (126,268)
								-_2_		-_1_			+_2_	+_3_	+_1_			+_5_	+_4_	-_3_			*D. rerio* (41,264)
	-_3_		+_2_		+_4_			+_1_						+_5_	-_1_	+_3_	-_2_		-_4_			-_5_	*C. elegans *(79,958)
	-_5_		+_4_					+_3_	-_2_	+_2_				+_5_	-_1_	+_1_	-_3_		-_4_		-_6_		*C. briggsae *(74,178)
										-_1_													*A. gambiae *(7,930)
+_1_						+_3_		-_1_		-_3_				+_2_			-_2_				-_4_		*D. melanogaster *(48,933)
+_1_		-_3_	-_2_			+_4_		-_1_			-_4_				+_3_		+_2_			-_5_	-_6_		*A. mellifera *(45,426)
																	+_1_	+_3_	+_2_				*A. thaliana *(109,900)
																							*S. pombe *(2,403)
																							*S. cerevisiae *(417)

*Indices signify rank order of slope coefficients, separately for negative and positive trends. ^†^L2, R2, S2 and L4, R4, S4 signify the two-fold and four-fold degenerate blocks of leucine, arginine, and serine, respectively. ^‡^*S. cerevisiae *terminal exons were retained given the small number of genes with more than one intron (eight).

But are these trends a peculiarity of mammals or common in other taxa? Does the presence or absence of trends correspond to what is known about the significance of exonic splicing regulation in each species? For example, a recent survey of several eukaryote genomes showed the SR protein family to be greatly expanded in metazoans but scarcely represented in unicellular genomes [13]. A failure to find preference trends in *S. cerevisiae*, an organism lacking SR proteins [14], might corroborate the hypothesis that preference patterns are indeed caused by ESEs. Moreover, if there are discernible trends in other species, do we repeatedly see the same amino acids avoided or preferred or are trends largely unique to each species? Also, are mammals unusual in showing a tight correlation between 5' and 3' trends, and may divergent results bear implications for the workings of the splicing machinery? Finally do we find more skews in species that *a priori *are expected to have a harder time identifying exons, that is, those in which exons are relatively small islands in a sea of intronic sequence? Here we examine these issues with exon data from a diverse set of species.

## Results

### Preference trends are widespread in multicellular species

**Figure 1:**
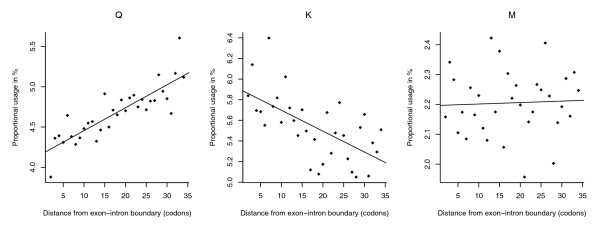
Nature and diversity of amino acid abundance trends near exon-intron boundaries. Relative abundance of glutamine (Q), methionine (M), and lysine (K) as a function of distance from the boundary across 5' ends of *D. melanogaster *exons is shown. Glutamine is significantly avoided near the boundary (rho = 0.86, *P* < 1.84E-7), lysine is preferred (rho = -0.65, *P *< 6.2E-5), whilst no significant trend is evident for methionine (rho = 0.096, *P* = 0.59). Note that a negative slope/rho value indicates a preference near the exon-intron boundary. Typically, where patterns of preference/avoidance are evident, we observe quasi-monotonic decreases/increases in relative abundance across the sequence range analyzed.

Exons from eight metazoan species (Human (Hs), mouse (Mm), *Danio rerio *(Dr), *Caenorrhabditis elegans *(Ce), *Caenorrhabditis briggsae *(Cb), *Anopheles gambiae *(Ag), *Drosophila melanogaster *(Dm), *Apis mellifera *(Am)), one plant (*Arabidopsis thaliana* (At)) and two ascomycetous fungi (*S. cerevisiae *(Sc), *Schizosaccharomyces pombe *(Sp)), were examined for trends in amino acid composition as one approaches the exon-intron boundary. Species were chosen from among a relatively small set of organisms for which high quality comparative data on splice-regulatory proteins have recently become available [13]. As splice site signals can extend into exons and our focus is on exonic splicing regulation, we removed the first full codon at the exon-intron boundary (see Materials and methods). Thereafter, rank correlations (rho) between distance from the boundary (34 codons into the exon; see Materials and methods) and proportional usage of the amino acid were computed independently for 5' and 3' regions of exons. Further, for all amino acids independently we fitted a linear regression extracting the slope of the line to be used as a crude diagnostic for the strength of amino acid preference/avoidance. Figure 1 illustrates the different types of relationship observed.

Two-fold and four-fold blocks of the six-fold degenerate amino acids were considered as distinct groupings so that a total of 46 tests (23 amino acid groups 5' and 3') were carried out for each species. Tables 1 and 2 give a comprehensive by-species overview of amino acid preferences/avoidances, significant after Bonferroni correction (N = 46 comparisons, *P *< 0.0011). Additional data file 1 contains the complete set of rank correlations for all 11 species.

The most conspicuous feature of Tables 1 and 2 is arguably the commonality of trends in the metazoa and the scarcity of trends in the ascomycetous yeast species. The two-fold block of leucine (L2) in *S. cerevisiae *is the only amino acid grouping exhibiting a significant preference trend (rho = -0.4482, *P *< 0.0003). This is in stark contrast to the suite of multicellular eukaryotes where an extensive range of avoidance and preference trends is observed. Only three multicellular species display fewer than 13 significant trends (Dm, Ag, At) whereas five (Hs, Mm, Ce, Cb, Am) display more than 20. For *D. melanogaster *and *C. elegans*, we tested whether the results might be biased as a result of exon homology, but in either case found amino acid abundance patterns at exon ends to be virtually identical in a set of homology-reduced genes (Dm, N = 8,840; Ce, N = 11,790; Additional data files 2 and 3).

The role of exonic guidance in splicing organization has been linked to multiple aspects of genome composition and pre-mRNA structure, including intron/exon length [15,16], intron number [7] and density [17] and splice site information content [7,18,19]. The number of significant amino acid trends per species tightly covaries with some of these factors, notably the mean number of introns per gene (rho = 0.95, *P *< 0.0001), median coding sequence (median CDS) per gene (rho = -0.97, *P *< 0.0007), genomic number of introns (rho = 0.86, *P *< 0.003), and intron length (log10(mean length): rho = 0.83, *P *< 0.006) as expected under a model where complex transcripts with multiple long introns elicit increasing reliance on exon definition [15]. On the other hand, neither SR protein family size (rho = 0.59, *P *= 0.09) nor splice site information content (5', rho = -0.26, *P *= 0.50; 3', rho = 0.43, *P *= 0. 25) show any relationship with the number of amino acid skews near intron-exon boundaries. The latter observation is perhaps the more interesting as it suggests that there is no straightforward compensatory relationship between splice site information content and the need for exonic regulation across species.

Finally, the number of exons from which amino acid trends were derived, although correlated with the number of trends (rho = 0.86, *P *< 0.003), does not feature among the top predictors when multicollinearity is controlled for (Additional data files 4-6). Together with the observation that we find relatively few trends in *Arabidopsis*, despite the substantial number of exons sampled, this suggests that sample size is not the critical factor in detecting different numbers of trends across species. We must stress, however, that the above results should be regarded as strictly exploratory given the small number of observations (Additional data file 4). A greater number of species with more comprehensive phylogenetic sampling will be required to validate the results in the future.

The preeminence of exon-intron structure in predicting the number of amino acid trends suggests that the intron-poor ascomycetous fungi analyzed here might not be representative of their kingdom. We therefore analyzed the composition of exon ends in *Cryptococcus neoformans *(Cn), an intron-rich basidiomycete. Strikingly, we find a large number (26) of preference and avoidance trends in this species (Table 3 and Additional data file 1), with some marked similarities in comparison to metazoan trends, particularly 5'. Furthermore, the inclusion of *C. neoformans *data in the analysis of potential predictor variables does not substantially change previous results: the mean number of introns per gene (rho = 0.91, *P *< 0.0002), median CDS per gene (rho = -0.68, *P *< 0.032) and the genomic number of introns (rho = 0.72, *P *< 0.02) remain strong predictors (Additional data file 6).

**Table 3:** Amino acids significantly preferred (-) or avoided (+) at 3' (top rows) and 5' (bottom rows) exon ends of *C. neoformans *compared to human

Amino acids^*†^																							
	
A	C	D	E	F	G	H	I	K	L4	L2	M	N	P	Q	R4	R2	S4	S2	T	V	W	Y	Species (number of exons)
+_3_		-_7_		-_3_			-_2_	-_1_		-_5_		-_6_	+_2_		+_1_	-_4_		+_4_					Human (178,438): 3'
	-_6_		+_1_	-_2_	+_4_	-_7_	-_1_	+_3_	-_3_					+_6_	-_5_	+_2_		+_5_				-_4_	*C. neoformans *(28,446): 3'
																							
+_2_			-_4_	-_5_		+_7_	-_3_	-_1_		-_2_	-_8_	-_6_	+_1_	+_4_	+_3_	-_7_		+_5_	+_6_				Human (178438): 5'
	-_9_			-_5_	-_7_		-_3_			-_1_	-_6_		+_2_		+_3_	-_4_	+_1_			-_8_	-_10_	-_2_	*C. neoformans *(28,446): 5'

*Indices signify rank order of slope coefficients, separately for negative and positive trends. ^†^L2, R2, S2 and L4, R4, S4 signify the two-fold and four-fold degenerate blocks of leucine, arginine, and serine, respectively.

Virtually nothing is known about the splicing mechanism in *C. neoformans *but the demonstration of alternative splicing pathways in this species [20] as well as low splice site information content (Additional data file 5) [7] make the presence of exonic splicing regulation a credible possibility. Consistent with this, the predicted *C. neoformans *proteome contains multiple proteins resembling known eukaryotic SR proteins, particularly in that they harbor RNA recognition domains (Additional data file 7). This is suggestive of involvement in splicing, albeit evidently insufficient to reach conclusions about specific functional roles of these proteins.

### Cross-species patterns

Whilst the spectra of amino acids preferred/avoided by individual species are ultimately unique in breadth (how many trends) and composition (which amino acids are affected), there is considerable cross-specific overlap in terms of whether a particular trend is present at all, its direction, and relative strength (as measured by the slope of the line of best fit). Tables 1 and 2 illustrate that this particular agreement is virtually perfect between human and mouse [2], with marginal differences in the relative strength of individual trends, and that directionality is conserved throughout. Considering zebrafish (Dr) as the only other vertebrate in our sample alongside these species, we notice that its spectrum is slightly diminished in breadth and contains a few trends not seen in the two mammals (G (3'), V (5',3')). However, overall concordance in composition and strength is still remarkably good, and the 'mammalian pattern of directionality' perfectly adhered to. The nematode pair almost matches the human-mouse dyad in terms of overall concordance of preference patterns, with directionality perfectly conserved.

For the most part, the patterns of preference/avoidance are repeatable across species. Table 4 shows pairwise comparisons between species giving rank correlations (rho) for the slopes derived from all 23 amino acid groupings. For the vertebrate group both 5' and 3' correlations are very high (all rho > 0.9, all *P *< 1.81E-06; 90 tests, significance threshold, *P *< 5.56E-04), with human and mouse in almost perfect agreement. More remarkably, however, some strong correlations also exist 3' between the vertebrates and, for example, *Anopheles *(all rho > 0.87, all *P *< 2.94E-06) and *Drosophila *(all rho > 0.75, all *P *< 2.9E-05). The 3' correlations are less impressive for the remaining species (Am, At, Cn) but *Apis *(all rho > 0.75, all *P *< 4.11E-05) and even *Cryptococcus *(all rho > 0.69, all *P *< 5.56E-04) boast remarkably strong 5' correlations with the vertebrates. Focusing on specific amino acid trends, isoleucine (I) stands out in that it is strongly preferred near 3' boundaries across all species; others are well represented, albeit not universal, through the entire phylogeny - for example, 5' avoidance of glutamine (Q), and 3' preference for phenylalanine (F).

**Table 4:** Cross-species correlations of preference slope coefficients considering all 23 amino acid groupings, 5' (bottom-left) and 3' (top-right)^*†^

	Hs	Mm	Dr	Ce	Cb	Ag	Dm	Am	At	Cn
Hs	1	0.99^++^	0.93^++^	0.71^++^	0.67^++^	0.88^++^	0.84^++^	0.53^+^	0.11	0.08
Mm	0.99^++^	1	0.92^++^	0.69^++^	0.67^++^	0.88^++^	0.85^++^	0.60^+^	0.20	0.15
Dr	0.92^++^	0.90^++^	1	0.74^++^	0.71^++^	0.87^++^	0.77^++^	0.48^+^	0.16	0.14
Ce	**-0.43^+^**	**-0.39**	**-0.40**	1	0.98^++^	0.84^++^	0.72^++^	0.37	0.24	0.16
Cb	**-0.60^+^**	**-0.56**	**-0.65^+^**	0.78^++^	1	0.82^++^	0.71^++^	0.34	0.21	0.17
Ag	0.62^+^	0.60^+^	0.61^+^	0	**-0.26**	1	0.89^++^	0.50^+^	0.18	0.18
Dm	0.64^+^	0.61^+^	0.51^+^	**-0.04**	**-0.14**	0.64^+^	1	0.57^+^	0.21	0.15
Am	0.76^++^	0.79^++^	0.77^++^	**-0.32**	**-0.41**	0.48^+^	0.46^+^	1	0.66^+^	0.55^+^
At	0.44^+^	0.44^+^	0.50^+^	**-0.36**	**-0.36**	0.06	0.19	0.40	1	0.75^++^
Cn	0.72^++^	0.69^++^	0.75^++^	**-0.31**	**-0.53**^+^	0.39	0.21	0.54^+^	0.52^+^	1

*S. pombe and *S. cerevisiae *omitted for clarity given the absence of significant correlations. ^†^Negative correlations in bold. +, significant at *P *= 0.05; + +, significant at *P *= 0.05/90 = 5.56E-04 (N = 90 tests).

### Deviant nematodes

**Figure 2:**
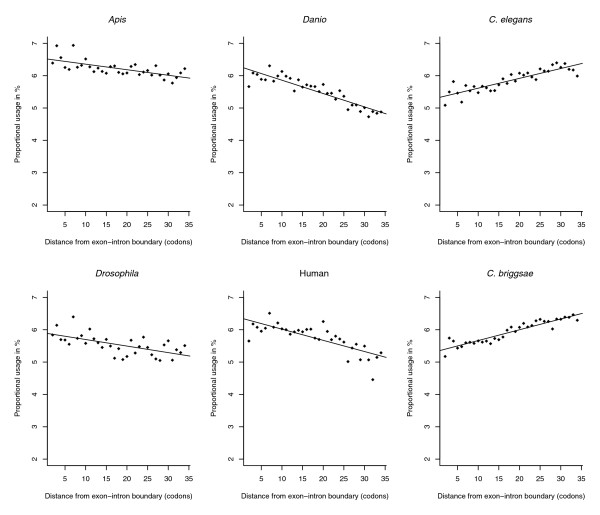
Relative amino acid abundance of lysine (K) at 5' ends of exons in six species. Proportional usage of lysine *vis-à-vis *all other amino acids is plotted against distance from the exon-intron boundary measured in amino acids. Variable degrees of preference for lysine near the boundary are evident for non-nematode species (Am, rho = -0.67, *P *= 2.71E-05, β(slope) = -0.017; Dr, rho = -0.79, *P *= 6.51E-07, β = -0.035; Dm, rho = -0.65, *P *= 6.11E-05, β = -0.020; Hs, rho = -0.90, *P *= 3.67E-09, β = -0.041) whereas nematodes show strong avoidance trends (Ce, rho = 0.89, *P *= 5.26E-08, β = 0.030; Cb, rho = 0.92, *P *= 0, β = 0.033).

The strong cross-species concordance in preference patterns makes one observation all the more striking. The nematode 5' spectra behave in a highly counterintuitive manner in that the 'mammalian pattern of directionality' is violated on several occasions: where we do find significant trends in nematodes and other species (E, K, L2, Q, R4, R2, T), all but glutamine (Q) show discrepant directionality (Table 2). For example, whereas lysine (K) is strongly preferred near boundaries in vertebrates and some insects (Dm, Am), it appears to be strongly avoided in the 5' region of nematode exons (Figure 2). Table 4 also underlines the exceptional position of nematodes: 5' correlations between nematodes and any other species are pervasively negative. No single correlation across all amino acids is significantly different from zero applying the adjusted significance threshold (*P *< 5.56E-04), owing to several trends collapsing into insignificance rather than fully reversing sign. However, the pervasiveness of this pattern is nonetheless noteworthy, especially considering that the same is not the case for the 3' spectra where we find a coherent agreement between nematodes and vertebrates (minimum rho > 0.65, all significant at *P *< 5.92E-04) and only the two-fold block of serine (S2) shows a reverse pattern of directionality among the significant trends for individual amino acids.

### Many species obey an approximately symmetric pattern of preference trends 5' and 3'

**Figure 3:**
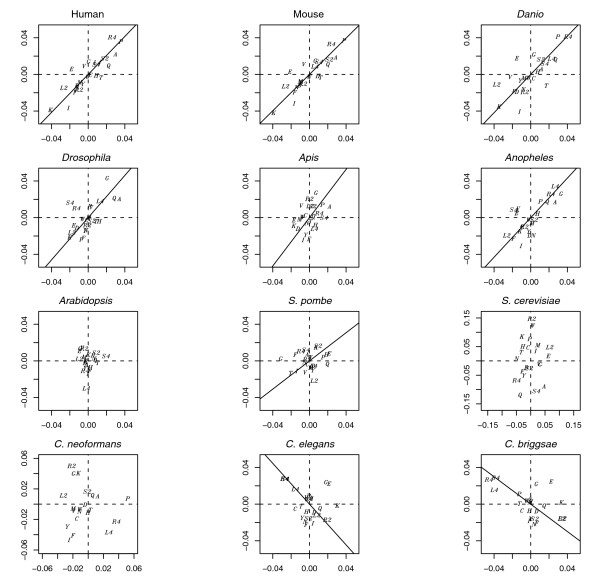
Variable symmetry in amino acid abundance trends comparing 5' and 3' exon ends within species. Intraspecific correlations between the 5' (x-axis) and 3' (y-axis) slopes as extracted from individually fitted linear models considering all 23 amino acid groupings are shown. Approximately symmetric arrangements are particularly evident for some species (notably vertebrates) whereas nematode arrangements (Ce, Cb) are not symmetric. Further notable is the higher variability of slope coefficients in some species (vertebrates and nematodes) *vis-à-vis *others (Am, At). Amino acids are represented by their one letter code (two-fold blocks are denoted by '2'). The regression lines are from SMA regressions. Lines were not fitted for *Arabidopsis*, *Cryptococcus *and *S. cerevisiae *given concerns about the adequacy of this technique for these datasets (see Materials and methods). For associated statistics consult Table 5.

This curious discrepancy between 5' and 3' spectra of amino acid trends in nematodes led us to investigate further the relationship of 5' and 3' patterns across species. Considering all amino acid trends simultaneously, rank correlations between slope coefficients (5'~3') were computed. Furthermore, we wanted to explicitly test the hypothesis that preference trends show a 'symmetric' behavior, that is, that individual amino acids exhibit preference trends of similar strength and direction at 5' and 3' ends. To this end, we carried out standardized major axis regressions (SMA; see Materials and methods) [21,22] for 5' versus 3' trends in each species and compared the resulting regression line with one expected under perfect symmetry (y = x). The results are given in Table 5 and graphically represented in Figure 3. Human and mouse show very substantial positive correlations between 5' and 3' preference trends (Hs, rho = 0.8528, *P *= 1.96E-06; Mm, rho = 0.8626, *P *= 2.28E-06). Although diminished in strength, we also see significant correlations for *Drosophila *and *Danio*. As expected from the previous analysis, correlations for nematodes are negative, albeit not significantly so (Ce, rho = -0.1413, *P *= 0.5185; Cb, rho = -0.4358, *P *= 0.0388). However, the SMA results allow us to reject any notion of *C. elegans *or *C. briggsae *adhering to a symmetric pattern of amino acid usage, the respective confidence intervals (CIs) ruling out a symmetry slope of β = 1 (CI (Ce), [-1.118; -0.7309]; CI (Cb), [-0.7474; -0.5139]). No other species for which an SMA could be carried out (Table 5; Materials and methods) deviate significantly from a symmetric model, although symmetry of amino acid trends varies greatly and can only really be called a defining characteristic of exon ends in vertebrates.

**Table 5:** Intraspecific 5'~3' correlations of preference slopes for all 23 amino acid groupings

			SMA		
			
	Rho	*P*-value*	Slope (β)	Lower Cl^†^	Upper Cl^†^
Human	0.85	1.96E-06	1.04	0.83	1.29
Mouse	0.86	2.28E-06	0.99	0.80	1.23
*D. rerio*	0.66	8.3E-04	1.04	0.78	1.40
*C. elegans*	-0.14	0.52	-1.11	-0.73	-1.69
*C. briggsae*	-0.44	0.04	-0.75	-0.51	-1.09
*A. gambiae*	0.57	5.16E-03	1.08	0.79	1.48
*D. melanogaster*	0.61	2.49E-03	1.15	0.82	1.62
*A. mellifera*	0.39	0.06	1.32	0.88	1.96
*A. thaliana*	-0.22	0.30	NA^‡^	NA^‡^	NA^‡^
*S. pombe*	0.22	0.31	0.77	0.50	1.17
*S. cerevisiae*	0.16	0.46	2.42^§^	1.58	3.70
*C. neoformans*	0.02	0.92	NA^‡^	NA^‡^	NA^‡^

*With 12 species significance is indicated by *P *= 0.05/12 = 4.17E-03. ^†^CI = 0.95, the regression line was forced through the origin. ^‡^See Materials and methods. ^§^Adequacy of SMA regression analysis is seriously in doubt for *S. cerevisiae *because normal distribution of residuals is strongly violated. NA, not available.

### Amino acid trends are largely consistent with participation in ESE motifs

Intriguingly, asymmetries in the amino acid composition of nematode exon ends appear to be mirrored by a corresponding asymmetry of regulatory motifs. Robinson [23], using a computational approach to characterize candidate ESEs in *C. elegans*, found that 5' and 3' ends were distinguished by different classes of consensus motifs. Crucially, he found purine-rich human-like candidate motifs to be associated with 3' ends but not 5' ends of nematode exons, which is broadly consistent with our observation that amino acids encoded by purine-rich codons tend to be, in contrast to other animals, disfavored at 5' ends (Table 2 and Figure 3).

For mammals, the prediction that amino acids preferred near boundaries should correspond to those favored in ESEs was tested by Parmley *et al*. [2]. The authors defined a metric that quantifies the involvement of amino acids in splice enhancer hexamers relative to the null expectation that every codon is represented in ESEs around its genomic frequency. As predicted, these hexamer preference indices (HPIs), computed for each amino acid grouping, were found to correlate with preference trends, strongly preferred amino acids on average associated with higher HPI values.

This relationship holds true for human as well as murine ESE sets and amino acid trends, considering either rank correlation coefficients (rho_x_; Hs HPI~rho_x_, rho = -0.54, *P *< 0.00001, N = 46; Mm HPI~rho_x_, rho = -0.49, *P *= 0.0005, N = 46) or the slope (β) of the fitted linear model (Hs HPI~β, rho = -0.57, *P *< 0.0001, N = 46; Mm HPI~β, rho = -0.52, *P *= 0.0002, N = 46).

As expected from the demonstration that ESEs can act at varying distances from the splice site [14], human ESEs do not exhibit a reading frame bias beyond what is expected from the genomic frequencies of the underlying codons (Additional data file 8). They can also, in principle, incorporate most codons (Additional data file 8). In consequence, the defined set of amino acids we find avoided or preferred are likely not due to ultimate exclusion of certain codons but because different efficacy and specificity across ESEs mean that often only a well-defined subset of codons can be used to specify the desired ESE.

Unexpectedly, when we derived HPIs for zebrafish amino acids, using a set of ESEs obtained from the same source [24], we found a significant correlation of reverse sign (Dr HPI~rho_x _(5'), rho = 0.6, *P *< 0.003, N = 46; HPI~rho_x _(3'), rho = 0.59, *P *< 0.0033, N = 46). Many experimentally verified ESEs have been characterized as A-rich and C-poor relative to the background frequency of these nucleotides in coding sequence. Whilst we found this to be the case for putative human ESE motifs not shared with zebrafish (A, 47.38% (ESE) versus 25.57% (exonic); C, 15.28% versus 25.99%, N(ESE) = 204), and for ESEs present in both species (A, 50% versus 25.57%; C, 6.37% versus 25.99%, N = 34), unique zebrafish ESEs (that is, ESEs not present in human) from this dataset were unusually enriched in C (39.47% versus 25.99%, N = 288) and relatively poor in A (18.40% versus 25.57%). Although one would expect ESE motifs to vary across taxa, the discrepancies are so pronounced as to sit awkwardly next to the substantial similarities in amino acid trends (Tables 1 and 2). One criterion used by the Burge group [25] to identify candidate ESE motifs was for such motifs to be more common near weak versus strong splice sites. Therefore, one possible explanation is that C-richness is a characteristic of zebrafish ESEs near weak splice sites but not generally, so that the predicted ESEs are not representative of ESEs across the zebrafish genome. Alternatively, comparatively lower quality of the, then recent, zebrafish genome build might be responsible for the divergent results. A re-examination of these putative zebrafish ESEs with an updated genome build may be worthwhile.

### Reduced rates of evolution near the exon-intron boundary in species where ESEs are essential components of the splicing machinery

**Figure 4:**
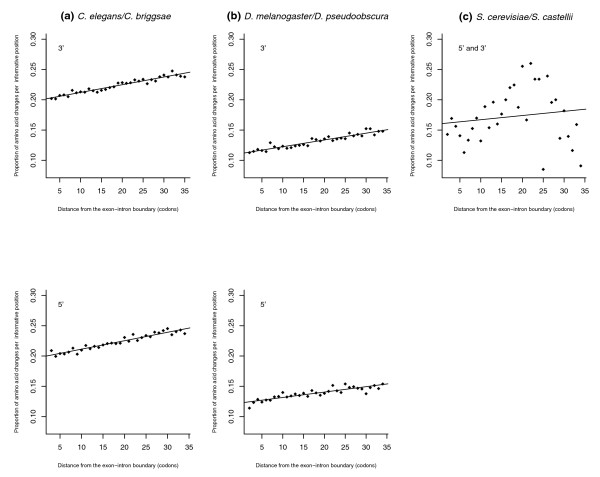
Frequency of nonsynonymous change as a function of distance from the exon-intron boundary. Amino acids are significantly more likely to be conserved near the exon-intron boundary comparing **(a) ***C. elegans*-*C. briggsae *(5', rho = 0.957, *P *= 0; 3', rho = 0.96. *P *= 0; N = 19,347 exons) and **(b) ***D. melanogaster*-*D. pseudoobscura *(5', rho = 0.87, *P *= 1.02E-07; 3', rho = 0.95, *P *= 0; N = 7,545 exons). The trends appear approximately monotonous and linear. Location-dependent conservation levels also appear slightly higher near the boundary comparing **(c) ***S. cerevisiae*-*S. castellii *but this is not significant (5', rho = 0.11, *P *= 0.55, N = 51; 3', rho = 0.11, *P *= 0.55, N = 39; pooled 3'/5', rho = 0.12, *P *= 0.51, N = 90) or of comparable monotony (but see Additional data file 9).

To further advance the hypothesis that gradients in amino acid abundance near exon-intron boundaries are a critical feature of exon ends in metazoans, we examined the degree of amino acid conservation as a function of distance from the boundary. For three pairs of species (*S. cerevisiae*-*Saccharomyces castellii*, *D. melanogaster*-*Drosophila pseudoobscura *(Dps); *C. elegans*-*C. briggsae*) sets of orthologous internal exons were derived from various sources and aligned at the amino acid level (see Materials and methods). Mirroring results from a comparison of human-mouse orthologues [2], we found strong and highly significant positive correlations of strikingly linear character (Figure 4) between distance from the boundary and amino acid substitution rate for the *Drosophila *and *Caenorhabditis *pairs, whilst proximity to the boundary did not appear to confer a higher level of amino acid conservation in the *Saccharomyces *comparison. Restricting the analysis to exons of at least 70 codons in length, we obtained qualitatively equivalent results (Drosophilae 5', rho = 0.53, *P *< 0.002, N = 3,690; Drosophilae 3', rho = 0.77, *P *= 9.70E-07, N = 3,690; Caenorhabdites 5', rho = 0.74, *P *= 2.33E-06, N = 6,273; Caenorhabdites 3', rho = 0.58, *P *= 4.5E-04, N = 6,273). This restriction ensures that all exons contribute an approximately equal share of information to each codon position from the boundary and eliminates the potential confounder that short exons might, for reasons unrelated to splicing, feature more frequently in highly conserved genes and create misleading trends by virtue of their disproportionate contribution to substitution rate information closer to the boundary.

Given that the set of aligned *Saccharomyces *exons consisted entirely of terminal exons (see Materials and methods), we repeated the analysis for a set of 5,352 orthologous pairs of terminal exons from our *Drosophila *dataset in order to rule out that differences are caused by any special characteristics of terminal exons. Correlations observed for terminal exons closely resemble those for internal exons (5', rho = 0.83, *P *= 3.8E-07; 3', rho = 0.75, *P *= 1.95E-06), alleviating any such concerns.

The above results appear consistent with greater functional significance of boundary-proximal amino acid composition in metazoans, proposed to be at least in part owing to their more extensive utilization of exonic splice regulatory sequences. However, after repeated (k = 10,000) random sampling of 90 aligned terminal exons from the *Drosophila *dataset and subsequent statistical analysis, we cannot reject the possibility that the *Saccharomyces *statistics were sampled from the same underlying distribution (Additional data file 9), implying that differences in conservation near exon-intron boundaries cannot be ultimately established from the data at hand.

**Figure 5:**
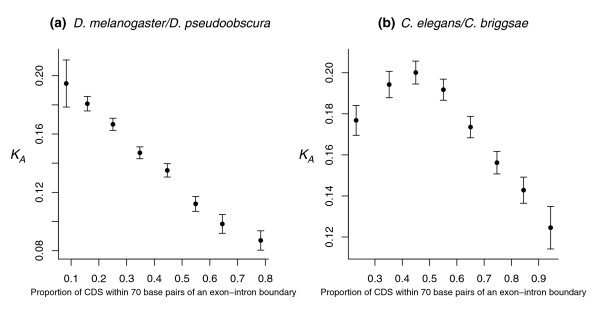
The rate of nonsynonymous evolution correlates negatively with the proportion of boundary-proximal sequence. *K*_*A *_is plotted as a function of the proportion of coding sequence located within 70 bp of an exon-intron boundary for **(a) ***D. melanogaster-D. pseudoobscura *orthologous genes (rho = -0.26, *P *= 2.2E-16, N = 4,132) and **(b) ***C. elegans*-*C. briggsae *orthologous genes (rho = -0.08, *P *= 6.18E-09, N = 5,248). The data have been divided into bins along regular decimal intervals (0.1, 0.2, and so on) and the mean *K*_*A *_within each bin plotted against the mean proportion of sequence near the boundary. The last (a) and first (b) three bins, respectively, have been pooled to obtain approximately equal bin sizes. Negative trends are present for both sets of aligned genes, but a departure from the general trend is evident for nematode genes with a low proportion of boundary-proximal sequence.

Having detected higher levels of amino acid conservation near exon-intron boundaries, we expect genes with a high proportion of sequences near boundaries ('flank-heavy') to evolve more slowly. This is indeed what we found when we considered *K*_*A *_as a function of the proportion of sequence within 70 bp of the boundary (Drosophilae, rho = -0.26, *P *= 2.2E-16, N = 4,132; Caenorhabdites, rho = -0.08, *P *= 6.18E-09, N = 5,248; Figure 5). We report *K*_*A *_rather than *K*_*A*_/*K*_*S*_, more commonly used as a measure of selection on protein sequence, because the underlying premise of *K*_*A*_/*K*_*S*_, namely that *K*_*S *_reflects neutral rates of evolution, is violated for sequence encoding ESEs [26].

The results are not qualitatively affected by contracting (50 bp) or expanding (100 bp) the region considered to constitute the boundary flank (Additional data file 10). Focusing on the terminal bins in Figure 5a, it appears that between *D. melanogaster *and *D. pseudoobscura *a gene with less than 10% of coding sequence near an exon-intron boundary evolves, on average, almost twice as fast (mean *K*_*A *_= 0.195) as a gene with more than 70% of boundary-proximal sequence (mean *K*_*A *_= 0.099). Discrepancies in evolutionary rate between 'flank-heavy' and 'core-heavy' bins appear less marked for the nematode pair (mean *K*_*A *_(%CDS near boundary >0.9) = 0.12; mean *K*_*A *_(%CDS near boundary <0.3) = 0.18). However, Figure 5b suggests that this is principally owing to curiously elevated levels of conservation for genes with a small proportion of sequence near the boundary, that is, genes with very large exons, a feature we did not encounter in the analysis of either insect (Dm-Dps) or mammalian (Hs-Mm) orthologues [2].

**Figure 6:**
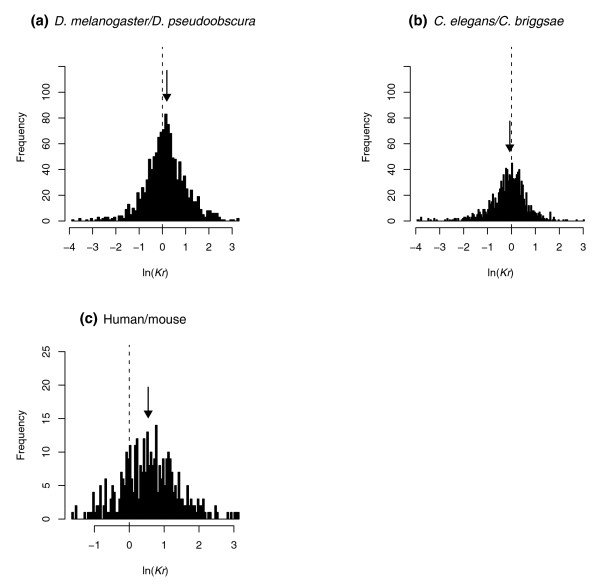
Exon cores and flanks evolve at different rates. Histograms of logged *Kr *ratios (*K*_*Ac*_/*K*_*Af*_), using 100 bins, for **(a) ***D. melanogaster-D. pseudoobscura *orthologous genes (N = 1,237), **(b) ***C. elegans-C. briggsae *orthologous genes (N = 1,102), and **(c) **human-mouse orthologous genes (N = 360) with a minimum of 300 bp of concatenated middle and flanking sequence of exons are plotted. The dashed line in each graph indicates ln(*Kr*) = 0, the point at which middle and flanking sections evolve at the same average rate. The arrows indicate the median logged *Kr *ratios of (a) 0.128, (b) -0.065, and (c) 0.559, respectively. All three are significantly different from the null expectation of ln(*Kr*) = 0 (*P *< 0.0001). Note the much more marked departure from the null expectation in the mammalian dataset.

Importantly, this anomaly highlights a more general reservation, namely that any measure capturing the proportion of sequence near the boundary will strongly covary with exon length, which in turn might covary with underlying functional determinants of evolutionary rate entirely unrelated to splicing control. Thus, in order to control for any putatively distorting effects of functional class on *K*_*A*_, we employed the following strategy: For each aligned gene, we concatenated the flanking regions of all exons, 5' and 3', defined as the first 72 bp bordering the exon-intron junction of trimmed exons. By implication, genes with no exon larger than 144 bp had to be excluded from this analysis. Concurrently, we concatenated the core sections of all exons of sufficient length in the respective gene, defined as the sequence block enclosed by the two flanking regions. As accurate estimation of *K*_*A *_probably requires a minimum of 100 codons, we further restricted analysis to those genes with at least 300 bp in the concatenated flanks and in the concatenated cores of exons. For each gene meeting the above criteria we then determined the rates of amino acid evolution in the concatenated core sections (*K*_*Ac*_) and flanking sections (*K*_*Af*_). We find that more *Drosophila *orthologous genes than expected by chance have faster evolving core regions (median (*K*_*Ac *_- *K*_*Af*_)/*K*_*Af*_) = 0.14, Wilcoxon signed rank test *P *< 0.0001, N = 1,237; Figure 6), consistent with the evidence, presented above, for additional sequence constraint operating on flanking regions. A significant tendency towards more rapid evolution in core sections is also evident when we confine the sample to genes with at least 600 bp in flanking as well as core regions (median (*K*_*Ac *_- *K*_*Af*_)/*K*_*Af*_) = 0.14, Wilcoxon signed rank test *P *< 0.0001, N = 785). Despite exhibiting the expected shift towards average higher *K*_*A *_in the core of exons, this trend is much less pronounced than in a previously reported comparison of human-mouse orthologues (median (*K*_*Ac *_- *K*_*Af*_)/*K*_*Af*_) = 0.68, Wilcoxon signed rank test *P *< 0.0001, N = 360; Figure 6c, and see Parmley *et al*. [2] for details). Curiously, for the nematode pair, we find significant evidence for a reverse correlation (300 bp, median (*K*_*Ac *_- *K*_*Af*_)/*K*_*Af*_) = -0.07, Wilcoxon signed rank test *P *< 0.0001, N = 1,102; 600 bp, median (*K*_*Ac *_- *K*_*Af*_)/*K*_*Af*_) = -0.014, *P *< 0.038, N = 496), that is, in the majority of genes, flanking regions evolve at a marginally higher rate than core regions.

## Discussion

### General trends

Parmley *et al*. [2] recently presented evidence that, in mammals, amino acid usage in the vicinity of exon-intron boundaries is affected by factors unrelated to protein function but to sequence-based information required for correct splicing. The objective of the present study was to elucidate whether such requirements have left an evolutionary imprint on exonic sequence composition across a phylogenetically diverse set of species. To this end, we systematically compared trends in relative amino acid abundance near exon-intron boundaries in 12 eukaryotic species. Our analysis revealed that preference for or avoidance of certain amino acids near boundaries is a common phenomenon among metazoan species but is not unique to metazoans. More amino acids show skewed usage in species where a greater problem identifying intron-exon boundaries is to be expected, that is, those with large and numerous introns. Notably, this includes the basidiomycete *C. neoformans*, suggesting that exonic splicing regulation might be a generic characteristic of species with complex pre-mRNA structures rather than absent from the fungal kingdom by virtue of phylogeny. Preference patterns show unmistakable signs of conservation along several dimensions: composition, relative strength, and directionality. The concordance in directionality (whether an amino acid is preferred or avoided) is particularly impressive in that we observe many commonalities with the mammalian pattern even in only distantly related species.

We do not claim that the systematic patterns we observe are solely caused by a selected preference for codons involved in ESEs. In fact, composite trends are almost certain to be the result of multiple functional constraints, including the need to avoid intron-specific enhancer motifs (for example GGG in mammals [25]) as well as motifs that would disrupt exon recognition. Furthermore, abundance trends could partially be the result of cryptic splice site avoidance as suggested by Eskesen and colleagues [27]. However, many of the trends observed - for example, cytosine avoidance near boundaries - are not predicted by this model [2,11].

Introns associate non-randomly with the codon in direct proximity to the splice site in a phase-specific manner, an observation often described as insertional preference [28]. Trimming and elimination of the first full codon should guard against picking up such insertional preferences or an extended splice site consensus. We cannot rule out that some boundary-proximal codons have slipped into our dataset owing to poor splice site annotation. However, it must be pointed out that this reservation applies only to the subset of amino acid trends that show biased usage directly adjacent to introns and might be more relevant to the interpretation of local discontinuities (Additional data file 11). Also, if the above-mentioned explanations were of major relevance, we would expect cryptic splice site avoidance, insertional preference, and (to a lesser extent) poor splice site annotation to cause similar patterns in ascomycetous yeasts, in particular *S. pombe*, for which a dataset of reasonable size is available. This is not the case.

Establishing to what extent these trends are caused by preference for ESEs will ultimately depend on characterizing species-specific catalogues of ESE/Exonic splicing silencer (ESS) motifs together with their corresponding *trans-*factors and relating these to the observed spectra of preferred/avoided amino acids. This work, in particular relating to tissue- and stage-specific splicing patterns, is still in its infancy [29], the catalogues currently available restricted to a small number of vertebrates and yet to be fully verified experimentally [30,31].

However, the dearth of significant trends in *S. cerevisiae *and *S. pombe *strengthens the proposition that preference trends principally reflect requirements to accommodate exonic splicing regulators. Although the *S. cerevisiae *genome codes for an SR protein kinase (Sky1p) with the capacity to phosphorylate mammalian arginine-serine rich (RS) domains, the likely endogenous substrate (the SR protein-like Npl3p) does not appear to be involved in pre-mRNA splicing [3,32]. Importantly, no splicing factors homologous to metazoan SR proteins have been discovered in *S. cerevisiae *[14], consistent with the classical view that splicing in budding yeast is regulated intronically. This is further consistent with the observation that splice site consensus is generally highly conserved, especially 5', much more so than in other species, including *C. neoformans *(Additional data file 5). The fact that our analysis revealed a significant 3' trend for the two-fold block of leucine (L2) might hint at the presence of recognition motifs in yeast exonic sequence. However, at present there is no evidence supporting the regular involvement of an ESE-like binding motif in *S. cerevisiae *splicing and alternative explanations should be considered.

Splicing in *S. cerevisiae *is moderately common in quantitative terms because many highly expressed genes, notably encoding ribosomal proteins, contain introns, so that over 25% of the mRNA population are spliced [33]. However, in over 6,000 *S. cerevisiae *genes we find less than 300 introns in total, so that splicing can hardly be considered a processing stage representative on a genome-wide scale. In contrast, splicing is much more prevalent in *S. pombe *where approximately 40% of genes contain introns [34]. Basal splicing proteins show an enhanced similarity to their mammalian homologues and two SR protein homologues (Srp1p, Srp2p) have been identified [35-37]. Unlike in budding yeast, there is recent evidence that Srp2p binds to specific exonic elements and interacts with the fission yeast orthologue of human splice factor U2AF [38]. Why then, given that SR protein-ESE-like interactions seem to exist in *S. pombe*, do we not find any trends for amino acid or codon preference in this species? We suggest that trends may be lacking for two reasons. Firstly, given the comparatively low level of splice site consensus degeneracy, a minimal number of ESEs might be sufficient to ensure correct splicing. On a genomic level, we might then fail to register biased abundance patterns on the spatial scale investigated in this study. Secondly, for clear-cut preference trends to evolve, a minimum level of splice-regulatory complexity might be required. This fits with our observation that more amino acid trends are observed in species with complex, intron-rich gene structures, including the yeast *C. neoformans *(Additional data file 6). Further, alternative splicing contexts, where regulatory elements frequently compete for precedence if arranged close to each other, could be envisaged as an evolutionary pressure initially driving the diversification of ESEs and corresponding *trans-*factors, thereby creating an environment in which strong trends might be required to attract or repel the correct set of *trans-*factors, both for constitutively and alternatively spliced genes. Consistent with this hypothesis, reports of alternative splicing in *S. cerevisiae *[39] and *S. pombe *[40] are restricted to singular cases, for which functionality of the recovered alternative splice products remains to be shown [41]. However, attempts to link diversity and density of ESEs to alternative splicing have so far yielded ambiguous results [42].

The absence of preference patterns in ascomycetous yeasts has an important practical implication. Finding amino acid trends to be abundant near exon-intron boundaries can be regarded as evidence for exon-based splicing regulation, without prior knowledge of specific binding motifs or *trans*-factors, although failure to detect such trends is insufficient to rule out interaction between exons and auxiliary proteins in the splicing process (compare *S. pombe*).

### Nematode exceptionalism in an ESE framework: is *trans*-splicing to blame?

The fundamental deviation from the 'mammalian pattern of directionality' shown by the 5' amino acid trends in nematode exons (Table 1) might, at first sight, be unexpected. There are extensive homologies between vertebrate and nematode basal splicing machineries on the protein level [13]. Furthermore, splicing in SR protein-depleted cells of the *Caenorrhabditis *relative *Ascaris lumbricoides *can be rescued by adding SR proteins derived from non-nematode (HeLa) whole cell extracts, supporting at least a minimum degree of functional overlap [43]. Thirdly, the high level of conservation between SR and SR-like proteins identified in each species explicitly includes the RNA recognition motifs, tentatively suggesting similar binding specificities [44].

There is, however, one feature of the nematode splicing process that sets it apart from the other species in our sample: a substantial proportion (approximately 70%) of *C. elegans *(and *C. briggsae*) genes are *trans-*spliced [45]. In this process a short (22 nucleotide) 5' small nuclear RNA (snRNA) fragment, the spliced leader, which is transcribed from a different genomic locale, is added at the 5' end of the pre-mRNA [46]. It would, we suggest, be highly disadvantageous for this *trans*-splicing machinery to act at the 5' end of exons where *cis*-splicing should occur. Indeed, were *trans*-splicing to occur where intron removal should take place, a gene would, in effect, be broken in two. Thus, we suggest that 5' ends of internal exons have evolved to ensure that they do not attract the *trans*-splicing machinery. Given that this machinery is ubiquitous in a cell, all 5' ends of internal exons, be they from *trans*-spliced genes or not, should be equally under pressure to avoid *trans*-splicing where *cis*-splicing should happen. Consistent with this expectation, the trends seen at 5' and 3' ends in internal exons are the same in genes from operons and those not in operons (data not shown). Interestingly, information content at 3' splice sites in nematodes is strikingly higher than in other species (Additional data file 5), as previously observed [47], further supporting the idea that splicing regulation in nematodes is unusual in its asymmetry.

What might be the proteins involved in *trans*-splicing? There is good evidence that several stages of the *trans*-splicing process are, like *cis*-splicing, critically supported by SR proteins [43,48]. Furthermore, whilst mammalian and *Ascaris *SR protein extracts are equally efficient in catalyzing *cis*-splicing *in vitro*, *Ascaris *SR protein extracts engender an approximately five-fold higher *trans-*splicing activity [43]. Although the use of whole cell extracts in these experiments precludes an analysis of the differential contribution of individual SR proteins, these observations are consistent with the hypothesis that a subset of splice-regulatory proteins in these species is dedicated to *trans-*splicing.

Given the above, we envisage *trans*-splicing specific SR and other proteins to interact primarily with intergenic sequence upstream of the first exon of the pre-mRNA to provide further guidance for the *trans*-splicing apparatus or mediate other functions crucial to *trans*-splicing, such as protecting downstream RNA from degradation [45,49]. A prediction derived from this model is that we should find in nematodes proteins participating in *trans*-splicing that bind to nucleotide motifs depleted of codons from amino acids avoided near the 5' end of exons.

### Symmetric exons?

Owing to their deviant 5' trends, nematodes stand out in another aspect of systematic amino acid biases. Parmley *et al*. [2] observed no significant differences in preference trends between 5' and 3' ends of exons in mammals. Similarly, approximate symmetry has been reported for ESE distribution in human exons [30]. Conversely, standardized major axis regressions [21,22] strongly suggest that nematodes do not conform to a symmetric pattern of preference trends.

An assessment of this situation very much depends on how we expect ESE-guided splicing regulation to work on a mechanistic level. If SR proteins are assumed to interact directly with specific components of the basal splicing machinery, as is probably the case for U2AF [3], we would not automatically expect the same ESEs (and by implication amino acid trends) to be represented at similar frequencies 5' and 3' where different spliceosomal proteins are present. Predictions of whether symmetry might be of functional relevance, however, especially for scenarios of indirect interaction, cannot be derived from the data at hand.

Confidence intervals in our exploration of symmetry are large so that we cannot ascertain that symmetry is a dominant pattern throughout our species sample. However, some best estimates of SMA slopes (β) are tantalizingly close to perfect symmetry (Mm, β = 0.9907; Hs, β = 1.0362; Dr, β = 1.0439; Ag, β = 1.0788; Table 5), warranting more detailed examination of this potentially functional signature in the future.

### Patterns of amino acid evolution

Consistent with the proposition that trends in relative amino acid abundance are functionally important, we observe lower rates of nonsynonymous evolution near exon-intron boundaries in insects (Dm-Dps), nematodes (Ce-Cb) and mammals (Hs-Mm), indicative of higher selective constraint in this region. Furthermore, the proportion of coding sequence that is located near boundaries is a partial predictor of *K*_*A *_(Figure 5). Genes with a higher share of sequence partaking in exon flanks tend to show reduced rates of evolution. Nematode genes, again, stand out in that they do not conform to the negative linear relationship between *K*_*A *_and flank-heaviness found in other species pairs (Hs-Mm and Dm-Dps), but show unexpectedly high levels of conservation for genes with very large exons. The causes for this currently remain elusive. Similarly, we would not have predicted that in worms gene-specific differences between evolutionary rate in the flanking and core sections of exons are biased (if only slightly) towards more rapid evolution of flanking regions. However, the distribution of core-flank evolutionary rate differentials in worms appears comparable to the one for flies, a higher median evolutionary rate of core regions in the latter notwithstanding (Figure 6). Human-mouse orthologous genes on the other hand show a much more dramatic distributional shift towards faster evolution in exon cores (see distributions in Figure 6). Between-taxa differences in gene composition, especially relating to the presence of more and longer introns in mammals, might account for these differences: on a speculative note, information necessary to distinguish an exon from surrounding non-coding sequence might require a unique degree of conservation under these circumstances, perhaps severely restricting the leeway for nonsynonymous changes to occur in flanking regions. Alternatively, restrictions imposed by our experimental set-up, especially relating to minimum sequence length requirements, might have resulted in the selection of gene sets with divergent splicing characteristics in the different species pairs. We leave a closer dissection of these questions to further analysis.

## Conclusion

Biased usage of amino acids in the vicinity of exon-intron boundaries is a common feature in metazoan genes, with the direction of biases largely consistent between taxa. That the biases accord with sequence preferences of SR proteins and that such biases are not seen in intron-poor yeasts support the view that dual coding of DNA in exons, to specify both which amino acids to employ and where introns are to be removed, is a common feature of metazoan species and more generally in genomes in which exons are relatively small islands in a sea of intronic sequences in the immature mRNA. Interestingly, similar skews in amino acid composition can be observed for the intron-rich fungus *C. neoformans*, suggesting that exonic splicing regulation might occur in this species. In nematodes, the possible relationship between *trans*-splicing and the exceptional departure from the mammalian pattern of amino acid trends at the 5' end of exons deserves further scrutiny. The results presented here suggest a simple sequence-based, species-independent diagnostic for the relative importance of exonic splicing regulation in a particular species given nothing more than a well-annotated genome.

## Materials and methods

### Relative amino acid abundance near exon-intron boundaries

For 12 species (human, mouse, zebrafish, *C. elegans*, *C. briggsae*, *A. gambiae*, *D. melanogaster*, *A. mellifera*, *A. thaliana*, *S. cerevisiae*, *S. pombe*, *C. neoformans*) we established individual exon datasets derived from a small number of databases (Additional data file 12). Pre-established CDS tracks were followed in all but three cases (At, Sp, Cn), for which annotated chromosome/scaffold sequences were downloaded from the relevant database and exons extracted subsequently. Exons with identical locus IDs were then sorted into individual files, only retaining files with at least one internal exon. All locus files were subsequently checked to ensure coding sequence started with ATG, finished with a stop codon (TAA, TAG, TGA), had no internal stop codons, and was a multiple of three nucleotides. Locus files where one of the above prerequisites was violated were removed from the final dataset. We also eliminated exons containing one or more ambiguous nucleotides ('n'). The remaining exons were trimmed so that the first nucleotide was the first nucleotide of the first complete codon and the last nucleotide the last of the final complete codon. Then, we discarded all terminal exons to obtain the final exon sets. Gene models from which exons were derived are provided in Additional data file 13.

After splitting individual exons in half to ensure that no codon featured in both 5' and 3' analyses, we considered the trend in usage of each amino acid as a function of the distance from the boundary up to a maximum distance of 34 codons. Importantly, the codon in direct proximity to the boundary was also eliminated.

We then calculated Spearman rank correlations (rho) between the distance from the boundary (5' or 3') and proportional usage of the amino acid (that is, in proportion to the number of residues at that given distance) for the remaining 33 data points for each species. The three six-fold degenerate amino acids we split into blocks of four and two (that is, 'S4' signifies, TCA, TCC, TCG and TCT, while 'S2' signifies AGC and AGT). In relevant circumstances, the two-fold and four-fold blocks were treated as separate amino acids, yielding a total of 23 amino acid groupings.

For each amino acid grouping independently we fitted unweighted linear models and extracted the slope of the regression line to be used as a basic measure of the strength of individual preference trends. Note that a negative rho/slope implies an amino acid that is preferred near boundaries and a positive rho/slope implies a tendency to be avoided. Unless otherwise stated, results are reported as significant only if they remain significant after correction for multiple testing (see Results for adjusted *P*-values).

For the most part, trends are approximately monotonic and linear and hence adequately captured by simple linear models. For certain amino acids, departures from linearity, some recurrent across species and typically highly localized, do exist however. Unusual U-shaped 5' trends for proline, originally noted for human and mouse by Parmley *et al*. [2], are also present in other species (Ce, Dr). Further, some amino acids, notably isoleucine and the two-fold block of leucine, are disproportionately preferred in direct proximity to the boundary (after trimming) at 3' exon ends in several species. 'Popping out' from otherwise linear trends (Additional data file 14), these patterns are perhaps caused by participation of the relevant codons in an extended splice site consensus relevant for U5 snRNA-mediated exon joining (see Additional data file 11 for a more detailed discussion of recurrent, locally confined preference/avoidance patterns and potential functional explanations). As a corollary of discontinuities more generally, comparative interpretation of slope coefficients as an index of relative strength ought to be done with care. In particular, our rank ordering of slopes derives its value from providing another dimension through which congruence in preference spectra can be asserted, rather than being easily translated into differential functional impact on a mechanistic level.

### Modifications in the analysis of *S. cerevisiae *exons

Given the small number of internal exons in *S. cerevisiae *(only eight genes have more than one intron), we decided to include terminal exons in the final dataset (417 exons) for this species. The one end of each terminal exon that did not border the intron was excluded. Otherwise, the removal of irregularities (internal stop codons and so on) proceeded as described above. Restricted sample size also indirectly prompted a re-examination of the results obtained from Spearman's rank correlations because the presence of multiple tied ranks led to concerns about the adequacy of this statistic. However, using the more appropriate Kendall's tau statistic did not return any qualitatively different results.

### Cross-species patterns in preference across all amino acid groupings

For 5' and 3' datasets independently, Spearman's correlations were computed between the previously derived slope coefficients of all 23 amino acid groupings for every possible metazoan species pair. Ninety tests (with the number of species N = 10, N^2-N = 90) were carried out and significance threshold adjusted accordingly (*P *= 0.05/90 = 5.56E-04). We initially included both yeast species in the analysis but, as expected from the absence of significant individual amino acid trends, we found no significant correlations for the global amino acid set (data not shown). No loss of relevant information is incurred whilst clarity of presentation is enhanced when these species are excluded from the analysis and, in particular, the accompanying table (Table 5).

### Comparison of orthologous exons

#### *S. cerevisiae*-*S. castellii*

A set of *S. cerevisiae-S. castellii *orthologous genes, based on a re-annotation of the *S. castellii *genome by Wolfe and colleagues, were obtained from the Yeast Gene Order Browser [50]. For each *S. cerevisiae *gene that contributed exons to our analysis of amino acid abundance, we checked whether a homologous *S. castellii *gene was present on the same positional track, the rationale being to compare true orthologues rather than outparalogues. If putatively orthologous gene pairs were found on both tracks, implying the retention of two post-genome duplication paralogues in both species, only the pair on track 1 was considered. This procedure yielded 164 orthologue pairs. *S. castellii *open reading frame structure downloaded from the same source was used to eliminate all *S. castellii *genes that lacked any introns, did not have a regular start or stop codon, or whose exon sequence was not a multiple of three nucleotides. Further discarding all genes with unequal exon number or unequal intron phase between species, 51 gene pairs (102 exons) remained. We further eliminated all exons shorter than eight amino acids in length as these were considered uninformative. After trimming (see above) codons were translated into amino acids and orthologous exons aligned using MUSCLE (version 3.6) [51]. After alignment, the first and last amino acid of each exon were removed. Exons were then split in half so that any one amino acid features exclusively in either 5' or 3' analysis. We then calculated the number of amino acid changes over the total number of informative (amino acid present in both species) sites for each amino acid position from the boundary, including only exon ends that bordered an intron (that is, only the 3' end for the first exon and only the 5' end for the last exon).

Spearman's and Kendall's rank correlations between distance from the boundary and the proportion of amino acids changed were computed for 5' and 3' ends separately. Given the small sample sizes for end-specific analyses (N(5') = 51, N(3') = 39), we also computed rank correlations for 5' and 3' ends pooled. Linear models were fitted for each analysis, weighting by the number of informative sites at distance x from the boundary.

#### *D. melanogaster*-*D. pseudoobscura*

A list of *D. melanogaster-D. pseudoobscura *orthologous genes was obtained from the Inparanoid database [52]. *D. pseudoobscura *exons were downloaded from the flybaseGene track on the UCSC genome browser [53] and sorted into files by gene locus, eliminating genes with irregularities as described above. Using the orthologue list we established a set of 4,165 orthologue pairs for which genes were present in the cleaned datasets of both species; 2,677 gene pairs (comprising 7,545 orthologous internal exon pairs, and 5,352 orthologous terminal exon pairs) remain after checking for equal exon number and intron phase. Trimming of exons, alignment and statistical analysis were carried out as described for *S. cerevisiae-S. castellii*. The 3' and 5' ends were considered for each internal exon, whereas only exon ends bordering an intron were included in the analysis of terminal exons.

#### *C. elegans*-*C. briggsae*

Each *C. elegans *locus file was translated into protein and queried against a database of all translated *C. briggsae *locus files using BLAST (blastp), and vice versa. Only reciprocal best hits with an expectation E ≤ 1 were retained. After checking for equal exon number and intron phase, 5,358 orthologous gene pairs (19,347 orthologous internal exon pairs) remained. Trimming and alignment were carried out as described above for *Drosophila*. Orthologues for all comparative species are given in Additional data file 13.

### Intraspecific 5'~3' correlations and symmetry analysis

Covering all 23 amino acid groupings Spearman's rank correlations were computed between 5' and 3' trends within each species (N = 12, *P *= 0.05/12 = 4.17E-03).

SMA regressions were computed in R using the SMATR package [21,22] applying standard confidence limits (95% CI). As symmetry of the type x = y was to be tested, the regression line was forced through the origin. SMA regression requires estimates of the slope of the regression line to have a consistently positive or negative sign so that the major and minor axes can be identified unambiguously. This is not the case for either *A. thaliana *or *C. neoformans*, which are hence not amenable to this type of analysis and were not included. Further, residual distribution for *S. cerevisiae *shows significant deviation from normality so that results for this species should be interpreted with care.

## Abbreviations

Ag, *Anopheles gambiae*; Am, *Apis mellifera*; At, *Arabidopsis thaliana*; Cb, *Caenorrhabditis briggsae*; CDS, coding sequence; Ce, *Caenorrhabditis elegans*; CI, confidence interval; Cn, *Cryptococcus neoformans*; Dm, *Drosophila melanogaster*; Dps, *Drosophila pseudoobscura*; Dr, *Danio rerio*; ESE, exonic splicing enhancer; ESS, exonic splicing silencer; HPI, hexamer preference index; Hs, human; Mm, mouse; Sc, *Saccharomyces cerevisiae*; SMA, standard major axis; Sp, *Schizosaccharomyces pombe*; SR protein, serine-arginine protein.

## Authors' contributions

TW compiled, processed, and analyzed the data. JLP participated in the HPI analysis and provided scripts. LDH conceived of and coordinated the study. TW and LDH wrote the paper. All authors read and approved the final manuscript.

## Additional data files

The following additional data are available. Additional data file 1 is a table giving the amino acid trends for all species and associated statistics. Additional data file 2 is a table giving amino acid trends and associated statistics for homology-reduced gene sets of *D. melanogaster *and *C. elegans*. Additional data file 3 contains the protocol for homology reduction of *C. elegans *and *D. melanogaster *orthologues. Additional data file 4 contains the protocol for covariate analysis of abundance trends. Additional data file 5 is a table listing covariates of amino acid trends by species. Additional data file 6 is a table giving by-species cross-correlations for covariates of amino acid trends. Additional data file 7 is a table listing best blast hits of SR proteins against *C. neoformans *genes and Pfam domain scores in those genes. Additional data file 8 contains an analysis of ESE positioning in relation to the reading frame. Additional data file 9 is a figure showing re-sampling distributions of evolutionary rates. Additional data file 10 is a table giving rank correlations between *K*_*A *_and the proportion of sequence near the exon-intron boundary. Additional data file 11 contains a detailed characterization of specific local discontinuities. Additional data file 12 is a table giving the sources of exon datasets. Additional data file 13 is a table giving the gene model IDs from which exons were derived. Additional data file 14 is a figure giving examples of locally discontinuous preference trends. Additional data file 15 is a table detailing local discontinuities across selected species.

## Supplementary Material

Additional data file 1 Amino acid trends for all species and associated statistics. Click here for file

Additional data file 2 Amino acid trends and associated statistics for homology-reduced gene sets of *D. melanogaster *and *C. elegans*. Click here for file

Additional data file 3 Protocol for homology reduction of *C. elegans *and *D. melanogaster *orthologues. Click here for file

Additional data file 4 Protocol for covariate analysis of abundance trends. Click here for file

Additional data file 5 Covariates of amino acid trends by species. Click here for file

Additional data file 6 By-species cross-correlations for covariates of amino acid trends. Click here for file

Additional data file 7 Best blast hits of SR proteins against *C. neoformans *genes and Pfam domain scores in those genes. Click here for file

Additional data file 8 Analysis of ESE positioning in relation to the reading frame. Click here for file

Additional data file 9 Re-sampling distributions of evolutionary rates. Click here for file

Additional data file 10 Rank correlations between *K*_*A *_and the proportion of sequence near the exon-intron boundary. Click here for file

Additional data file 11 Detailed characterization of specific local discontinuities. Click here for file

Additional data file 12 Sources of exon datasets. Click here for file

Additional data file 13 Gene model IDs from which exons were derived. Click here for file

Additional data file 14 Examples of locally discontinuous preference trends. Click here for file

Additional data file 15 Local discontinuities across selected species. Click here for file
