# Supplementary material for: Finding exonic islands in a sea of non-coding sequence: splicing related constraints on protein composition and evolution are common in intron-rich genomes
Source: Genome Biol. 2008 Feb 7;9(2):R29. doi: 10.1186/gb-2008-9-2-r29 (PMC2374712; doi:10.1186/gb-2008-9-2-r29)
Supplement: Additional data file 3 — Protocol for homology reduction of C. elegans and D. melanogaster orthologues. [file gb-2008-9-2-r29-S3.doc]

**Supplementary Document 1: Homology reduction**

For *C. elegans* and *D. melanogaster* respectively, all genes contributing exons to the main dataset were translated into protein and assembled in a database, which was then blasted against itself (blastp). Genes were considered to be homologous if they had an eigenvalue of 1 or lower. Putative homologues were assembled into clusters (with some clusters containing only a single member) and one gene chosen at random to represent its cluster. This procedure yielded sets of 11790 Ce and 8840 Dm genes that were free from internal homologues. Minor differences in amino acid spectra are owing to small changes in the P values of amino acids originally close to the significance threshold. Results from the analysis of boundary-proximal amino acid trends can be found in Additional data file 2.
