# Supplementary material for: Finding exonic islands in a sea of non-coding sequence: splicing related constraints on protein composition and evolution are common in intron-rich genomes
Source: Genome Biol. 2008 Feb 7;9(2):R29. doi: 10.1186/gb-2008-9-2-r29 (PMC2374712; doi:10.1186/gb-2008-9-2-r29)
Supplement: Additional data file 4 — Protocol for covariate analysis of abundance trends. [file gb-2008-9-2-r29-S4.doc]

**Supplementary Document 2: Covariate analysis**

*C. briggsae* and *A. mellifera* were excluded from this analysis because reliable data for SR protein family size and other parameters were not available.

The pooled (5’ + 3’) number of significant amino acid abundance trends (T) was taken to reflect he ubiquity of exonic splicing regulation in the respective genome and was preferred to comparing slope parameters across species because magnitude of slope coefficients was adjudged to be more volatile to sample size. Generally, sample size is too low to generate a robust model of how the measures below are related to each other so the results should be seen as strictly exploratory. The following measures were initially considered as potential predictors of T:

| **Predictor** | **Obtained through/from…** |
| --- | --- |
| Number of (internal) exons used to derive trends [transformation: log10] | See Material and Methods |
| Size of SR protein family   - With candidates labelled dubious - Without dubious | Barbosa-Morais et al. 2006 [13] |
| Genomic number of introns | Gene models [see Additional data file 13], hereafter referred to as Set G, were obtained from the sources given in Additional data file 6 and genes filtered for likely protein-coding capacity as described in Material and Methods. The number of introns was then computed as a simple count, including single-intron genes, irrespective of splice consensus. |
| Genomic fraction of genic sequence that is intronic | Set G was used. The genomic fraction of genic sequence that is intronic is the sum of all intronic over all genic sequence (including intronless genes) |
| Intron density per kb CDS | From Carmel et al. (2007) [54], validated against Set G (R^2^=0.94) |
| Introns per gene (mean, median, IQR) | Set G was used. |
| Information content (IC)   - 5’splice site (-3 to +6) - 5’ss (+3 to +6) - 3’ss (-6 to +3) - 3’ss (-6 to -3)   (ss at +1|-1 (3’) or -1|+1 (5’) respectively) | IC was computed for all intron-containing genes from Set G with at least 3 flanking exonic nucleotides either side of the intron and minimum intron size of 20 nucleotides according to the formula  *IC=2+pA*log2(pA)+ pC*log2(pC)+ pG*log2(pG)+ pT*log2(pT)*  for the nucleotide ranges indicated. The more confined range was used by Irimia et al. (2007) [7] but we added an extended range, as splice-relevant information need not be confined to intronic sequence. |
| Intron length (mean, median, IQR)  [transformation: mean: log10; IQR: log10] | From intron-containing genes of Set G. |
| Exon length (mean, median, IQR) | From intron-containing genes of Set G. |
| CDS length (mean, median, IQR) | Set G was used. |
| Gene length (mean, median, IQR)  [transformation: median: log10] | Set G was used. |
| CDS per gene (mean, median, IQR) | Set G was used. |

Spearman rank correlations were computed for all measures versus T. As this analysis was regarded as exploratory, a lenient significance threshold was chosen (P<0.05) for predictors to be considered for further analysis. The weaker covariate of the two measures of central tendency (mean, median) was eliminated. All remaining predictors were linearized by transformation (log10) if necessary, checked for normal residual distribution, outliers, and homoscedasticity. No further eliminations/transformations were deemed necessary. Additional data file 5 contains all predictors retained. Predictors were then submitted to a mixed stepwise linear regression model. Probabilities to enter/exclude terms were set to 0.1. To prevent overfitting, only predictors that showed significant leverage (P<0.05) when entered alongside stronger predictors were accepted in the final model.

The number of observations is very small (9) and multicollinearity is high [see Additional data file 6], a problem for both multiple regression and principal component approaches. Inherent subjectivity of the latter made us pursue a regression approach, but it needs to be stressed that the reported results should be regarded as strictly exploratory and that validation will require a substantially larger number of species, sampled from a more comprehensive phylogeny.

For the analysis including *C. neoformans*, exactly the same procedure was followed except that “Size of SR protein family” could not be included as a predictor as information was not available for this species. Multiple regression results can be found in Additional data files 5&6.
