# Supplementary material for: Finding exonic islands in a sea of non-coding sequence: splicing related constraints on protein composition and evolution are common in intron-rich genomes
Source: Genome Biol. 2008 Feb 7;9(2):R29. doi: 10.1186/gb-2008-9-2-r29 (PMC2374712; doi:10.1186/gb-2008-9-2-r29)
Supplement: Additional data file 8 — Analysis of ESE positioning in relation to the reading frame. [file gb-2008-9-2-r29-S8.doc]

**Supplementary Document 3: ESEs show no biases with regard to reading frame**

The identity of amino acids preferred or avoided near exon-intron boundaries might be biased as a result of

1. it being impossible for an amino acid to be incorporated in an ESE, considering the nucleotide composition of all ESEs or
2. the distribution of ESEs being non-random with regard to the reading frame

We can only meaningfully test for such potential biases in species where the nucleotide composition of ESEs is well known across all functional ESE motifs. The only cases where we approach such a situation are human and mouse [30] (Fairbrother datasets, see section e) in the main text).

As these two species are virtually indistinguishable as far as their respective ESE catalogues and amino acid trends are concerned, we only describe results for human ESEs here.

a.

A basic analysis of possible codon identity in 238 human ESEs, across all possible reading frames, reveals that none of these ESEs directly incorporates the following codons:

TTA (L2 – weakly preferred)

TAA (stop)

TAG (stop)

CCC (P – strongly avoided)

CCG (P – strongly avoided)

CAC (H – weakly avoided)

CGG (R4 – strongly avoided)

GGG (G – no trend but avoided in other species)

Evoking complementing bordering codons all these codons could, in principle, be incorporated in ESEs but the observation that, with the exception of TTA, the codons not present in ESEs are avoided near exon-intron boundaries suggests that these codons rarely, if ever, form part of an ESE motif. That stop codons are not frequently incorporated is not surprising. Thus, we conclude that the observed spectrum of amino acid trends owes, in part, to the fact that ESEs can only give rise to a limited number of codons. However, to observe diverse spectra of preference and avoidance we probably have to evoke differences across ESEs, i.e. whether ESEs can *de facto* incorporate most codons. Differences in ESE efficacy are well established [14] and differences in specificity, whilst relatively poorly explored on a genome-wide basis, could be substantial, at least for a subset of ESEs (cf. [43] on *trans*-splicing specific SR proteins in nematodes). We therefore propose that the preference spectra observed can be explained by typical nucleotide composition of ESEs in conjunction with constraints on how efficient and specific ESEs have to be specified.

b.

ESEs might have a biased distribution as regards their reading frame. For example, AAAGAT might predominantly occur in frame 0 (i.e. the translated codons are AAA and GAT). This is, in fact, likely to be the case given that the codons, combinations of which give rise to any one ESE, vary in frequency. In contrast, we cannot see a convincing *a priori* functional hypothesis that would favour placement of an ESE in a certain reading frame. ESEs are known to act at varying distances from the splice site [14] so that precise spatial positioning does not seem to be of utmost importance.

We nonetheless tested whether a simple codon frequency model can explain the distribution of ESEs. The set of 178438 human internal exons from which amino acid trends were derived were screened for the presence of ESEs from the Fairbrother dataset (see main text). The screening region was restricted to the 2^nd^ to 34^th^ codon to correspond to the region for which amino acid trends were described. We excluded all ESEs that would inevitably (without addition of neighbouring codons) give rise to a stop codon in one of the three reading frames (30) because reading frame biases are expected for this group. Any instance of one of the remaining 208 ESEs was assigned to either 3’ or 5’ boundary (equidistant ESE motifs were discarded) and the reading frame was noted.

The frame- and boundary-specific ESE motif counts were compared to a model under which codons that make up ESEs are paired randomly by their genomic frequency. Human codon frequencies were downloaded from kazusa.or.jp/codon/. For frame 0 a hexameric ESE is simply specified with the likelihood corresponding to the product of both codon frequencies, e.g. 24.44*10^-3 (AAA) * 21.78*10^-3 (GAT). For other frames the added frequencies of all codons (excluding stop codons) that could potentially give rise to the leading/lagging fragment of the ESE were multiplied, e.g. in frame 1 AAAGAT can in principle be specified by all codons ending in -A, plus codon AAG, plus all codons starting AT-. Figure A below plots expected versus observed counts for all 208 ESE motifs. The null model describes the data well, especially for frame 0, where we do not make a random assumption as to the identity of neighbouring codons. Furthermore, if we consider ESEs outside the prediction interval, we find them (and the amino acids they harbour) to be strongly reminiscent of the strongest purine-rich ESE motif but, importantly, very similar across frames (Table A below). This is consistent with no frame preference of ESEs and that differences in relative prevalence across frames chiefly reflect codon frequencies. However, what is also evident from comparing ESEs occurring more and less frequently than expected, is that contextual information (“disruptive C”) is important in whether a certain codon is likely to specify an ESE, especially for GAA (coding for glutamic acid (E)).

We conclude that frame preference of ESEs is well explained by genomic frequencies of those codons that make up the ESE. Consequently, the same ESEs do occur in different reading frames and therefore give rise to different amino acid biases.

**Figure A.** Expected frequencies versus observed motif counts of human ESE motifs (by frame) at exon-intron boundaries

|  |
| --- |


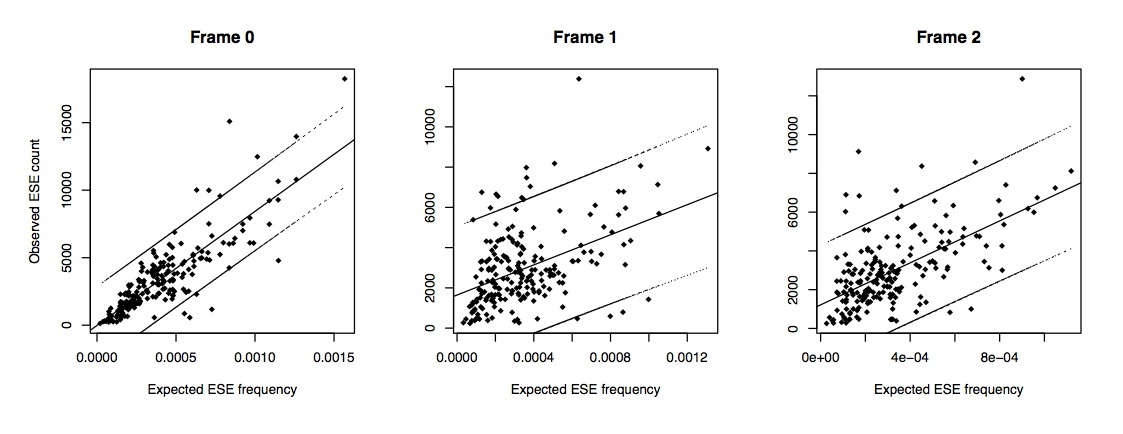


The solid line is the fitted linear model, the dotted lines delimit the confidence interval for the predicted values. The expected frequency derived from codon usage frequencies is a good predictor of the observed counts: Frame 0: Pearson’s r=0.85, P<2.2E-16; Frame 1: Pearson’s r=0.45, P<1.14E-11; Frame 2: Pearson’s r=0.60, P<2.2E-16

**Table A.** ESEs outside 95% prediction interval of linear model in Figure A

|  |
| --- |

| Frame 0 | | Frame 1 | | Frame 2 | |
| --- | --- | --- | --- | --- | --- |
| O>E | O<E | O>E | O<E | O>E | O<E |
| GAGGAG | AACGAA | A**GAA**GA | C**GAA**GA | AG**AAG**A | AC**GAA**A |
| GAGAAG | TTCGAA | A**GAA**AA | C**GAC**GA | AG**AAA**A | AC**GAA**G |
| AAGAAG | GACGAA | A**GAA**GC | C**GAA**AA | GG**AAG**A |  |
| GAAGAA | GAACTG | A**GAA**GG | C**GAA**CA | AG**AAT**G |  |
| AAGAAA |  | A**GGA**GA |  | GG**AGA**A |  |
| AAAGAA |  | T**GGA**AA |  | GA**AGG**A |  |
| GAAGAT |  | A**GCA**GA |  | GG**AGG**A |  |
|  |  | A**AGA**AA |  | GA**AGA**A |  |
|  |  | A**GGA**AG |  |  |  |
|  |  | T**GGA**AG |  |  |  |
|  |  | A**AGA**TG |  |  |  |
|  |  | A**AGA**AG |  |  |  |

O:observed; E:expected (compare Figure A); translated full codons are highlighted for frames 1 and 2
