# Supplementary material for: Finding exonic islands in a sea of non-coding sequence: splicing related constraints on protein composition and evolution are common in intron-rich genomes
Source: Genome Biol. 2008 Feb 7;9(2):R29. doi: 10.1186/gb-2008-9-2-r29 (PMC2374712; doi:10.1186/gb-2008-9-2-r29)
Supplement: Additional data file 9 — Re-sampling distributions of evolutionary rates. [file gb-2008-9-2-r29-S9.doc]

**Supplementary Figure 1.** Sampling distributions of statistics for a limited (N=90) number of aligned terminal exons from *Drosophila* dataset

|  |
| --- |


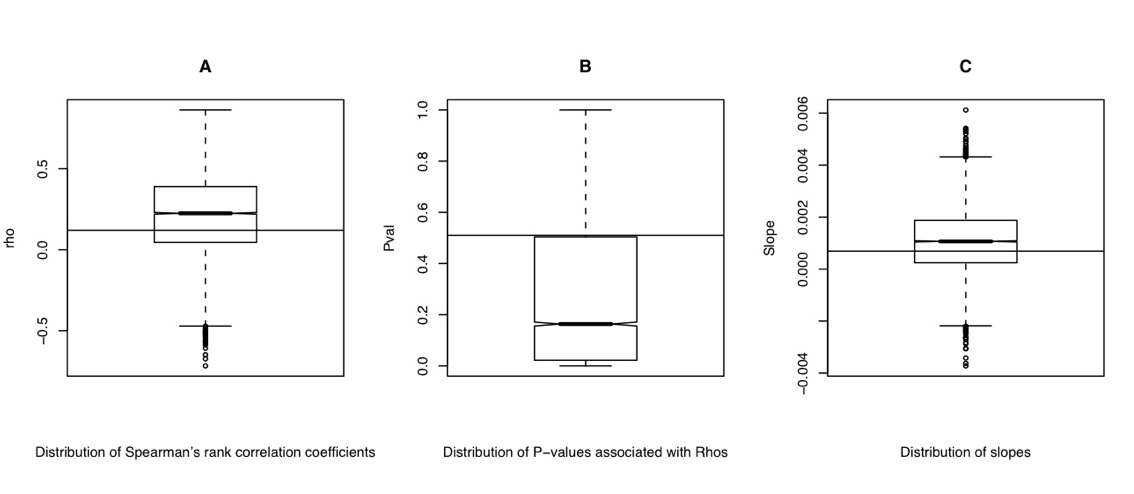


Supplementary Figure 1. To elucidate whether the correlative statistics obtained from the analysis of 90 aligned Saccharomyces exons differ from the other species chiefly because of the differences in exon numbers, we repeatedly (k=10000) sampled 90 aligned terminal exons from our Drosophila dataset (taking 39 first exons and 51 last exons to replicate the slightly unequal representation of these exon types in the Saccharomyces analysis), and obtained the core statistics for each sample as described in Methods. Distributions for (A) rho, (B) rho-associated p-values, and (C) slopes of the fitted linear are given above, the horizontal line indicating the values observed in the Saccharomyces analysis (rho=0.12, P=0.51, slope=0.00069). The plots indicate that – on average - we would expect to find a higher, more significant correlation, and a greater slope of the line of best fit than is the case for the Saccharomyces statistics, but also suggests that we cannot reject the possibility that these statistics are drawn from the same underlying distribution.
