# Supplementary material for: Finding exonic islands in a sea of non-coding sequence: splicing related constraints on protein composition and evolution are common in intron-rich genomes
Source: Genome Biol. 2008 Feb 7;9(2):R29. doi: 10.1186/gb-2008-9-2-r29 (PMC2374712; doi:10.1186/gb-2008-9-2-r29)
Supplement: Additional data file 10 — Rank correlations between KA and the proportion of sequence near the exon-intron boundary. [file gb-2008-9-2-r29-S10.doc]

**Supplementary Table 6** Rank correlation between K_A_ and proportion of sequence near the exon-intron boundary

|  |
| --- |

| **Species pair** | **Proportion within**  **x bp of the exon-intron boundary** | **Rho** | **P-value** |
| --- | --- | --- | --- |
| *D. melanogaster*  *– D. pseudoobscura* | x=50 | -0.26 | 2.2E-16 |
|  | x=70 | -0.26 | 2.2E-16 |
|  | x=100 | -0.26 | 2.2E-16 |
| *C. elegans*  *– C. briggsae* | x=50 | -0.078 | 1.79E-08 |
|  | x=70 | -0.080 | 6.18E-09 |
|  | x=100 | -0.080 | 6.14E-09 |
