# Supplementary material for: Finding exonic islands in a sea of non-coding sequence: splicing related constraints on protein composition and evolution are common in intron-rich genomes
Source: Genome Biol. 2008 Feb 7;9(2):R29. doi: 10.1186/gb-2008-9-2-r29 (PMC2374712; doi:10.1186/gb-2008-9-2-r29)
Supplement: Additional data file 11 — Detailed characterization of specific local discontinuities. [file gb-2008-9-2-r29-S11.doc]

**Supplementary Document 4: Characterization of local discontinuities**

Whilst fitting linear models to amino acid preference trends appears appropriate in most cases, there are a few recurrent patterns, some highly localized, that disturb linearity. Unusual U-shaped 5’ trends for proline, originally noted for human and mouse [2], are also present in other species (Ce, Dr). Where trends over a distance of more than 30 codons are recognizably non-linear, linear analysis can still reflect broad tendencies but fail to highlight divergent local patterns. And if such patterns reflect molecular mechanisms that are more narrowly defined in spatial terms, information crucial for a correct functional interpretation would be lost. This might be particularly pertinent for recurrent discontinuities concerning the second codon from the boundary (codon 2). Some amino acids are disproportionately preferred (I, L2, Y, F) or avoided (A) for codon 2, enhancing already existing preference trends in multiple species [see Additional data file 15; Figure S2A in Additional data file 14 for a typical example). Cook’s distances for several of these trends point to a disproportionate influence of these codons on the overall trend (data not shown). As a corollary, comparative interpretation of slope coefficients ought to be done with care. In particular, our rank ordering of slopes derives its value from providing another dimension through which congruence in preference spectra can be asserted, rather than being easily translated into differential functional impact on a mechanistic level.

The fact that some of these highly localized patterns are found across a number of only distantly related species suggests that paying attention to local discontinuities might reveal elements of functional significance.

Nonlinear enhancement of existing trends can be reconciled with the above-mentioned observation that efficacy of ESEs to catalyze splicing reactions declines with increasing distance from the boundary [9]. There is no reason why such declines have to be linear.

However, in the light of occasional preference/avoidance running counter to the background trend (e.g. Figure S2B in Additional data file 14) we can suggest an alternative explanation for this particular local bias: For the second transesterification reaction of the splicing process exons need to be aligned inside the spliceosome. U5 snRNA has been identified as the critical mediator of this alignment in yeast as well as metazoans [55, 56], initially contacting the 3’ end of the 5’ exon and subsequently bringing it into proximity to the 5’ end of the 3’ exon [55]. RNA-RNA interaction is reliant on base-pairing of exonic sequence with the invariant U5 snRNA loop 1, characterized by the 9 base pair consensus G_1_C_2_C_3_U_4_U_5_U_6_Y_7_A_8_Y_9_. It has been shown that positions 5 and 6 base-pair with positions –2 and –3 in the 5’ exon [57], leaving positions 7 to 9 as potential interactants for codon 2 (in phase 0 exons). Intriguingly, dissecting the contribution of individual codons to local trends for certain amino acids, we find that discontinuous preferences for codon 2 are wholly owing to those codons that offer a matching complement to the -Y_7_A_8_-consensus fragment (ie A/G_7_T_8_), namely TT_8’_A_7’_ and TT_8’_G_7’_ (L2) and AT_8’_A_7’_ (I), whereas amino acids are avoided owing to codons that contravene this complementation pattern, GA_8’_C_7’_, GA_8’_T_7’_ (D) and GC_8’_T_7’_ (A). Note that, moving from 3’ to 5’ into the exon, codons need to be read backwards.

The loop 1 consensus is perfectly conserved for all species featured in Rfam [58] including a representative coverage of the taxa in the present study (Sc, Sp, Dm, Mm, Hs, Ce, At). This is consistent with our finding that local trends for the affected amino acids are phylogenetically pervasive. However, to validate this hypothesis experimental proof will be required showing that a) base-pairing occurs between codon 2 and relevant positions (7,8) in the loop 1 consensus and b) that this process is functionally significant on a genomic level and can hence explain conservation across species.

Whether other local trends (F, Y, E) might be owing to the presence of yet uncharacterized splice-relevant motifs, functional requirements of protein composition, the avoidance of cryptic splice sites [11], or something else, we leave to further analysis.
