# Supplementary material for: Finding exonic islands in a sea of non-coding sequence: splicing related constraints on protein composition and evolution are common in intron-rich genomes
Source: Genome Biol. 2008 Feb 7;9(2):R29. doi: 10.1186/gb-2008-9-2-r29 (PMC2374712; doi:10.1186/gb-2008-9-2-r29)
Supplement: Additional data file 12 — Sources of exon datasets. [file gb-2008-9-2-r29-S12.doc]

**Supplementary Table 7** Sources of exon datasets

|  |
| --- |

| Species | Database (Website) | Track/Files | Number of internal exons in the final datasets (see Methods)* |
| --- | --- | --- | --- |
| Human | UCSC (http://genome.ucsc.edu/cgi-bin/hgTables) | RefSeq | 178438 |
| Mouse | UCSC (~) | RefSeq | 126268 |
| D. rerio | UCSC (~) | RefSeq | 41264 |
| C. elegans | UCSC (~) | RefSeq | 79958 |
| C. briggsae | UCSC (~) | Twinscan | 74178 |
| A. gambiae | UCSC (~) | Ensembl Genes | 7930 |
| D. melanogaster | UCSC (~) | RefSeq | 48933 |
| A. mellifera | UCSC (~) | NCBI genes | 45426 |
| S. cerevisiae | UCSC (~) | SGD Genes | 417 |
| A. thaliana | NCBI (ftp://ftp.ncbi.nih.gov/genomes/Arabidopsis_thaliana) | GenBank | 109900 |
| S. pombe | Sanger (ftp://ftp.sanger.ac.uk/pub/yeast/pombe/Chromosome_contigs) | Embl contigs | 2403 |
| C. neoformans | Stanford Genome Technology Center (http://www-sequence.stanford.edu/group/C.neoformans/files/) | sgtc | 28446 |

* for *S. cerevisiae* terminal exons were retained given the small number of genes with more than one intron (8)
