# Supplementary material for: Finding exonic islands in a sea of non-coding sequence: splicing related constraints on protein composition and evolution are common in intron-rich genomes
Source: Genome Biol. 2008 Feb 7;9(2):R29. doi: 10.1186/gb-2008-9-2-r29 (PMC2374712; doi:10.1186/gb-2008-9-2-r29)
Supplement: Additional data file 14 — Examples of locally discontinuous preference trends. [file gb-2008-9-2-r29-S14.doc]

**Supplementary Fig. 2** Examples of locally discontinuous preference/avoidance

|  |
| --- |


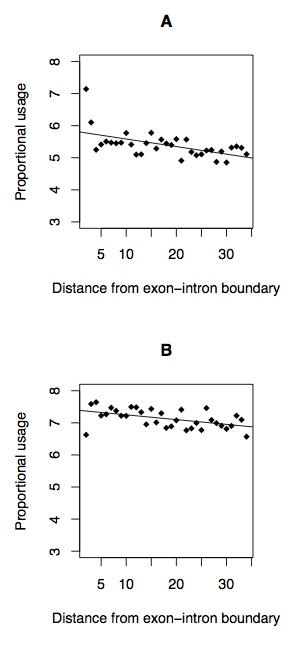


Discontinuities in the trends of relative amino acid abundance for the second codon from the boundary. Proportional usage of the respective amino acid is plotted as a function of distance from the exon-intron boundary. (A) Preference for isoleucine near 3’ boundaries in *Drosophila melanogaster*. Preference at the second codon position is disproportionately marked in relation to an otherwise linear trend. (B) Preference for glutamic acid near 5’ boundaries in *Danio rerio*. Abundance of glutamic acid at the second codon position runs counter to an otherwise linear preference trend, suggesting narrowly defined local avoidance of this amino acid.
