# Supplementary material for: Finding exonic islands in a sea of non-coding sequence: splicing related constraints on protein composition and evolution are common in intron-rich genomes
Source: Genome Biol. 2008 Feb 7;9(2):R29. doi: 10.1186/gb-2008-9-2-r29 (PMC2374712; doi:10.1186/gb-2008-9-2-r29)
Supplement: Additional data file 15 — Local discontinuities across selected species. [file gb-2008-9-2-r29-S15.doc]

**Supplementary Table 9** Patterns of discontinuous preference in direct proximity to the exon-intron boundary

|  |
| --- |

| Discontinuous avoidance (A) and preference (P) for the second codon from the boundary, enhancing (⇑) or running counter (⇓) to a significant global trend; () if trend not significant | | | | | | | | |
| --- | --- | --- | --- | --- | --- | --- | --- | --- |
| Y | D | I | F | E | L2 | A | Species |  |
|  | A() |  | P⇑ | A | P⇑ |  | 5’ - Human |
|  | A | P⇑ | P⇑ |  | P⇑ | A⇑ | 3’ |
| P() | A() |  | P⇑ | A | P⇑ |  | 5’ - Mouse |
|  | A | P⇑ |  |  | P⇑ | A⇑ | 3’ |
|  | A() |  | P() | A() | P⇑ |  | 5’ - D. rerio |
|  | A | P⇑ |  |  | P() | A() | 3’ |
| P⇑ |  |  |  | A⇑ |  | A⇑ | 5’ - C. elegans |
| P() |  | P⇑ | P⇑ |  | P() | A() | 3’ |
|  | A() | P() |  | A⇑ |  |  | 5’ - C. briggsae |
| P() |  | P⇑ | P⇑ |  | P⇑ | A() | 3’ |
|  |  |  |  |  |  |  | 5’ - A. gambiae |
|  |  |  |  |  | P⇑ |  | 3’ |
| P() |  |  | P() | A() |  | A⇑ | 5’ - D. melanogaster |
| P() |  | P⇑ | P⇑ |  | P⇑ | A() | 3’ |
| P() | A | P() |  | A |  | A⇑ | 5’ - A. mellifera |
| P⇑ | A() | P⇑ | P⇑ | A() | P | A() | 3’ |
|  |  |  |  |  | P() |  | 5’ - A. thaliana |
|  | A() | P⇑ |  |  | P() | A() | 3’ |
|  |  |  |  |  |  |  |  |
|  |  |  |  |  | P⇑ |  | 3’ – S. cerevisiae |
